# Contamination Effects on Improving the Hydrogenation/Dehydrogenation Kinetics of Binary Magnesium Hydride/Titanium Carbide Systems Prepared by Reactive Ball Milling

**DOI:** 10.3390/ma8105350

**Published:** 2015-10-10

**Authors:** M. Sherif El-Eskandarany, Ehab Shaban

**Affiliations:** Nanotechnology and Advanced Materials Program, Energy and Building Research Center, Kuwait Institute for Scientific Research, Safat 13109, Kuwait; eshaaban@kisr.edu.kw

**Keywords:** reactive ball milling, grain-growth inhibitors, FeCr contamination, hydrogen storage nanocomposites, kinetics, cycle-life-time

## Abstract

Ultrafine MgH_2_ nanocrystalline powders were prepared by reactive ball milling of elemental Mg powders after 200 h of high-energy ball milling under a hydrogen gas pressure of 50 bar. The as-prepared metal hydride powders were contaminated with 2.2 wt. % of FeCr-stainless steel that was introduced to the powders upon using stainless steel milling tools made of the same alloy. The as-synthesized MgH_2_ was doped with previously prepared TiC nanopowders, which were contaminated with 2.4 wt. % FeCr (materials of the milling media), and then ball milled under hydrogen gas atmosphere for 50 h. The results related to the morphological examinations of the fabricated nanocomposite powders beyond the micro-and nano-levels showed excellent distributions of 5.2 wt. % TiC/4.6 wt. % FeCr dispersoids embedded into the fine host matrix of MgH_2_ powders. The as-fabricated nanocomposite MgH_2_/5.2 wt. % TiC/4.6 wt. % FeCr powders possessed superior hydrogenation/dehydrogenation characteristics, suggested by the low value of the activation energy (97.74 kJ/mol), and the short time required for achieving a complete absorption (6.6 min) and desorption (8.4 min) of 5.51 wt. % H_2_ at a moderate temperature of 275 °C under a hydrogen gas pressure ranging from 100 mbar to 8 bar. van’t Hoff approach was used to calculate the enthalpy (∆*H*) and entropy (∆*S*) of hydrogenation for MgH_2_, which was found to be −72.74 kJ/mol and 112.79 J/mol H_2_/K, respectively. Moreover, van’t Hoff method was employed to calculate the ΔH and ΔS of dehydrogenation, which was found to be 76.76 kJ/mol and 119.15 J/mol H_2_/K, respectively. This new nanocomposite system possessed excellent absorption/desorption cyclability of 696 complete cycles, achieved in a cyclic-life-time of 682 h.

## 1. Introduction

Hydrogen storage is one of the key enabling technologies for realization of hydrogen energy economy [1]. Hydrogen storage materials, taking metal hydrides as a typical example, are commercially prepared by solvent-based synthesis methods or by direct gas–solid hydrogenation reactions. In contrast to the traditional gas–solid hydrogenation process, which is achieved at temperatures far above room temperature, an attractive method—so-called reactive ball milling (RBM) [2,3]—was developed in the 1990s to conduct the exothermic reactions between the gas- and metallic solid phases at almost room temperature. This relatively new process has been considered as a powerful tool for fabrication of different nanocrystalline metallic nitrides and hydrides [4]. In their room-temperature process, the starting metallic powders are subjected to dramatic shear and impact forces generated by the milling media (balls). The powders are, therefore, disintegrated into smaller particles with large surface area, and very clean or fresh oxygen-free active surfaces of the powders are created. The reactive milling atmosphere (nitrogen or hydrogen gases) was gettered and absorbed completely by the first atomically clean surfaces of the metallic ball-milled powders to react in a same manner as a typical gas–solid reaction [5]. Since then, the RBM process has become a common technique successfully used for preparing nanocrystalline metal hydrides, including magnesium hydride (MgH_2_) and their composite powders [1,6].

High capacity hydrogen storage materials such as MgH_2_ have been receiving much attention as promising solid-state hydrogen storage systems due to their high hydrogen storage capacity (7.60 wt. %), reversibility, cost effectiveness, availability and cyclability [7,8,9]. The international interest in the development of hydrogen based technologies, particularly the area of fuel cell electric vehicles, has greatly increased in recent years [9].

Unfortunately, and in contrast to the obvious advantages seen in MgH_2_ binary hydrogen storage systems, the high thermal stability and the difficulty to decompose this hydride system into metal and hydrogen gas, plus the poor hydrogenation and consequence dehydrogenation kinetics, lead to restricting utilization of such a light-weight system in real automobile applications [7,9,10].

Even though and in spite of the serious drawbacks found in MgH_2_, the worldwide interest in such an attractive binary metal hydride has been increased, especially after improving its hydrogen absorption and desorption kinetics by applying a longer ball milling time that led to destabilizing the β-MgH_2_ phase and increasing the volume fractions of the metastable γ-MgH_2_ phase [11]. Long mechanical ball milling time always is one key approach for releasing the crystalline stored energy, leading to refining the MgH_2_ grains along their grain boundaries resulting in a fine-grained structure. Such fine grains with their short-distance grain boundaries always facilitate a short diffusion path, allowing fast diffusion of the hydrogen atoms into the Mg lattice [12].

Moreover, ball milling the MgH_2_ with pure metallic catalysts (e.g., Ti, Fe, Ni, Nb, V) [13], intermetallic compounds (e.g., Zr_100−*x*_Ni*_x_*, and Ti-based alloys) [14,15,16], metal carbides such as TiC [17], metal oxides such as Nb_2_O_5_ [18], metal chlorides such as MgCl_2_ [19], rare earth chlorides such as LaCl_3_ [20], and nanocomposite Ni/Nb_2_O_5_ powders [21] led to remarkable improvement in the hydrogen absorption/desorption kinetics and lowering the thermal stability of MgH_2_. It has been shown by Ismail [20] that the improved hydrogen storage properties of MgH_2_ doped with LaCl_3_ were due to the catalytic effects of the La-Mg alloy and MgCl_2_. Such ultrafine micro-scaled/nano-scaled powders serving as catalysts have shown the possibility of improving the hydrogenation/dehydrogenation properties of MgH_2_ to open up a new horizon for its real application.

In the present study, we have investigated the effect of FeCr contamination introduced to the MgH_2_ powders upon ball milling in the long term on improving the hydrogenation/dehydrogenation properties of the metal hydride phase. Moreover, the effect of doping the as-synthesized MgH_2_ nanocrystalline powders with TiC nanopowders on the hydrogen storage capacity and cyclability of MgH_2_ was studied in terms of morphology and kinetics.

## 2. Experimental Procedure

Pure Mg metal powders (~80 μm, 99.8% purity provided by Alfa Aesar— Ward Hill, MA, USA), synthesized TiC nanopowders obtained upon high-energy ball milling of Ti and graphite powder (~100 nm, 2.4 wt. % FeCr), and hydrogen gas (99.999%) were used as starting materials. A certain amount of the Mg powders (5 g) was balanced inside a helium (He) gas atmosphere (99.99%)—glove box (UNILAB Pro Glove Box Workstation, mBRAUN, Garching, Germany). The powders were then sealed together with 50 FeCr- stainless steel balls into a FeCr steel vial (220 mL in volume), using a gas-temperature-monitoring system (GST; supplied by evico magnetic, Dresden, Germany). The ball-to-powder weight ratio was 40:1. The vial was then evacuated to the level of 10^−3^ bar before introducing H_2_ gas to fill the vial with a pressure of 50 bar. The milling process was carried out at room temperature using high energy ball mill (Planetary Mono Mill PULVERISETTE 6, Fritsch, Idar-Oberstein, Germany). After 200 h of RBM, the powders were discharged from the vial inside the glove box and sealed into two Pyrex vials. The as-synthesized MgH_2_ powders were then mixed in the glove box with the desired weight percentage (5%) of TiC, using an agate mortar and pestle. Five gram of the mixed powders were charged together with 50 hardened steel balls into the hardened steel vial and sealed under He gas atmosphere [21]. The vial was then filled with 50 bar of hydrogen gas atmosphere and mounted on the high-energy ball mill. The milling process was interrupted after selected time (25, and 50 h) and the powders obtained after an individual milling time were completely discharged into 8 Pyrex vials for different analyses. The average crystal structure of all samples was investigated by X-ray diffraction (XRD) with CuKα radiation, using 9 kW Intelligent X-ray diffraction system, provided by SmartLab-Rigaku, Tokyo, Japan. The local structure of the synthesized material powders at the nanoscale was studied by 200 kV-field emission high resolution transmission electron microscopy/scanning transmission electron microscopy (HRTEM/STEM) supplied by JEOL-2100F, Tokyo, Japan, equipped with Energy-dispersive X-ray spectroscopy (EDS) supplied by Oxford Instruments, Oxfordshire, UK. The morphological properties of the powders after selected ball milling times were determined by 15 kV-field emission scanning electron microscope (FE-SEM, JSM-7800F, Tokyo, Japan) equipped with EDS supplied by Oxford Instruments, UK. The concentrations of elemental Mg, Ti, Fe, and Cr in the as-ball milled powders were determined by inductively coupled plasma optical (ICP) emission spectrometry. Shimadzu Thermal Analysis System/TA-60WS, using differential scanning calorimeter (DSC), was employed to investigate the thermal stability indexed by the decomposition temperatures of MgH_2_ and to estimate the activation energy, using the Arrhenius approach with different heating rates of 7, 8, 9, and 10 °C/min. The hydrogenation properties, including absorption/desorption kinetics and cycle-life-time, were investigated via Sievert’s method, using PCTPro-2000, provided by Setaram Instrumentation, Caluire, France.

## 3. Results

**Figure 1:**
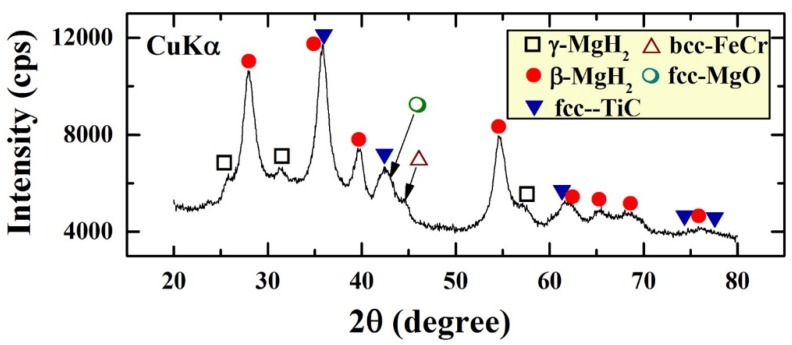
XRD patterns of MgH_2_ nanocrystalline powders obtained after 200 h of RBM time and then ball-milled with TiC powders for 50 h.

The XRD pattern of the end-product of MgH_2_/5.2TiC/4.6FeCr nanocomposite powders obtained after 50 h of ball milling is shown in Figure 1. The powders composed of β-MgH_2_ (PDF file #: 03-065-3365) and γ-MgH_2_ (PDF file #: 00-035-1184) phases mixed with fcc-TiC phase (PDF file #: 00-031-1400). This end-product was significantly contaminated (~2.3 wt. %) with bcc-FeCr alloy (PDF file #: 00-054-0331) introduced to the powders upon using FeCr stainless steel as milling tool. A significant amount of bcc-FeCr was obtained as shown in Figure 1. Moreover, handling the powders outside of the glove box led to a surface oxidation of the powders and the formation of magnesium oxide layers, as indicated by the Bragg-peaks belonging to fcc-MgO phase (PDF file #: 00-004-0829) shown in Figure 1. Obviously, the as-prepared nanocomposite powders revealed broad Bragg peaks, suggesting the formation of nanocrystalline grains.

**Figure 2:**
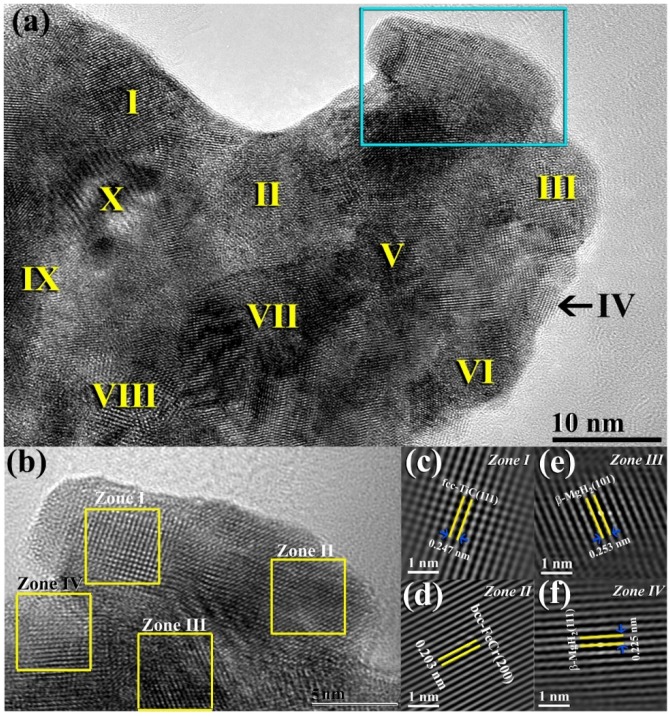
(**a**) BFI micrograph of as-milled MgH_2_/5.2TiC/4.6FeCr nanocomposite powders obtained after 50 h of ball milling time. The Roman Numerals presented in (**a**) refer to the points used for EDS local analysis (Table 1). The atomic-resolution TEM image of the squared zone indexed shown in (**a**) is presented in (**b**). The FFT lattice images for zones I, II, III, and IV, shown in Figure 2b are displayed in (**c**–**f**), respectively.

The bright field image (BFI) of nanocomposite MgH_2_/5.2TiC/4.6FeCr powders obtained after 50 h of ball milling is displayed in Figure 2a. The powders revealed Moiré-fringes of different phases. This is suggested by the dissimilarity in the interplanar spacing (2d), as shown in Figure 2a. The HRTEM image of the indexed square region shown at the edge of the powders in Figure 2a is presented in Figure 2b. The fast Fourier transform (FFT) patterns corresponding to the examined square regions presented in Figure 2b are displayed in Figure 2c,f. The atomic array with a long-range ordered structure that was presented in Zone I corresponding to nanocrystalline TiC grain. This was confirmed by the interplanar spacing of 0.247 nm (Figure 2c) that well matches with fcc-TiC (111). Zone II Figure 2b refers to the precipitation of bcc-FeCr contamination, as confirmed by the interplanar spacing of 0.203 nm for (200), as presented in Figure 2d. Zones III and IV display two individual regions in the MgH_2_ matrix corresponding to β-MgH_2_ (101) and (111), which are well matching with the interplanar spacing of 0.253 nm (Figure 2c) and 0.225 nm (Figure 2f), respectively.

The distribution of TiC/FeCr into the MgH_2_ matrix was examined by intensive EDS local analysis performed at selected points (Roman Numerals symbols shown in Figure 2a) and listed in Table 1. The results show that the concentration of TiC/FeCr is remarkably varied from one region to another beyond the nano-level, as shown in Table 1. It is worth mentioning that significant FeCr contamination was evident within those TiC-rich areas (II, IV, VII, X), as shown in Table 1. This is attributed to the existence of high FeCr contamination content in the as-prepared nanocrystalline TiC powders. However, a considerable amount of FeCr contamination content existed in the as-prepared MgH_2_ nanocrystalline powders, as can be seen in the rich Mg-area presented in Table 1 (I, V, VI, and VIII).

**Table 1:** Local EDS elemental analysis of the points presented in Figure 2a for MgH_2_/5.2TiC/4.6FeCr nanocomposite powders obtained after 50 h of ball milling. The oxygen content introduced to the sample during TEM sample preparations is not included.

Point	Elements (wt. %)					
	Mg	Ti	C	Fe	Cr	Total
I	97.03	1.60	0.38	3.42	0.57	100
II	15.65	40.30	10.12	29.21	4.72	100
III	95.79	0.73	0.18	2.83	0.47	100
IV	48.70	28.63	7.16	12.87	2.64	100
V	76.03	9.82	3.96	8.26	1.93	100
VI	97.29	0.88	0.22	1.38	0.23	100
VII	39.70	30.57	7.16	18.93	3.64	100
VIII	98.59	0.24	0.08	0.93	0.16	100
IX	97.20	1.13	0.26	1.18	0.23	100
X	61.2	18.65	4.83	12.79	2.53	100

**Figure 3:**
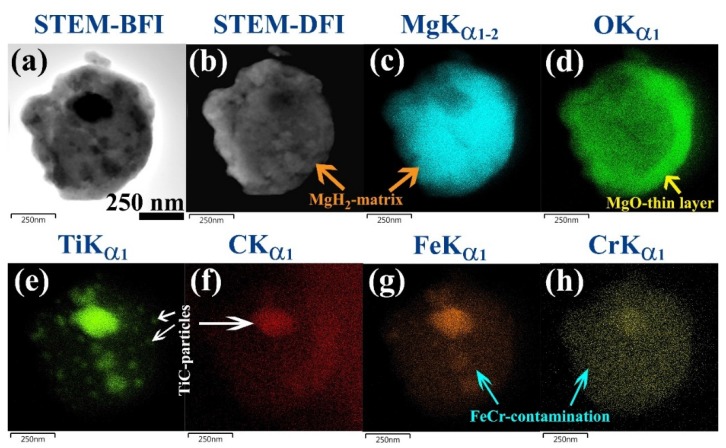
(**a**) STEM-BF; (**b**) STEM-DF micrographs and the corresponding X-ray elemental mapping of (**c**) Mg; (**d**) O; (**e**) Ti; (**f**) C; (**g**) Fe; and (**h**) Cr for aggregated MgH_2_/5.2TiC/4.6FeCr nanocomposite powders obtained after 50 h of ball milling.

In order to get more information about the TiC/FeCr distribution embedded into the host MgH_2_ matrix, STEM-EDS X-ray elemental mapping was performed. Figure 3 presents the images of STEM-(bright field) BF (a), STEM-(dark field) DF (b) and the corresponding EDS chemical mapping for Mg (c), O (d), Ti (e), C (f) Fe (g), and Cr (h) of an agglomerated powder obtained after 50 h of the ball milling. The powder had nearly a spherical-like morphology with a size of about 520 nm in diameter (Figure 3a). Obviously, the powder after this stage of milling had a rough surface topology related to attachment with TiC nanocrystalline particles (Figure 3b). As a result of SEM sample preparations and handling the powders outside of the glove box, the MgH_2_ powder (Figure 3c) was oxidized, as indicated by a thin-layer of MgO coat with a thickness of about 68 nm, as shown in Figure 3c. Nanocrystalline TiC (Figure 3a,e,f) was homogeneously distributed onto the surface of MgH_2_ powders. The individual TiC particle size was in the range of 10–20 nm in diameter, as shown in Figure 3e). However, some agglomerated TiC particles with apparent sizes ranging between 80 nm and 220 nm were bonded onto the MgH_2_ surfaces, as shown in Figure 3e. The FeCr contamination introduced to the powders upon using steel balls was homogeneously distributed in the MgH_2_ matrix, as elucidated in Figure 3g,h. We should emphasize that the concentration of FeCr contamination was higher in the regions containing TiC-particles when compared with the MgH_2_-matrix region, as shown in Figure 3e–h.

**Figure 4:**
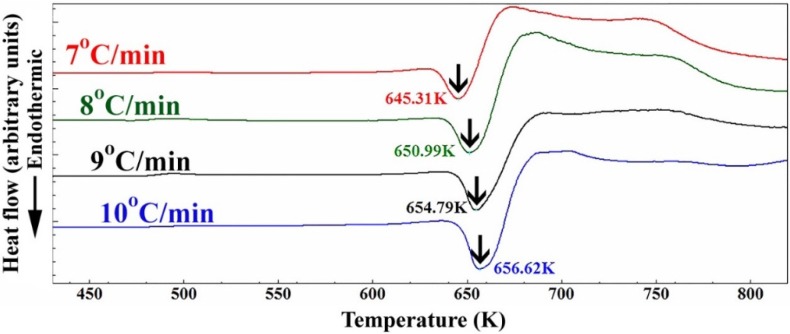
DSC curves achieved at different heating rates (7, 8, 9, and 10 °C/min) of nanocomposite MgH_2_/5.2TiC/4.6FeCr powders obtained after 50 h of milling.

The thermal stability of nanocomposite MgH_2_/5.2TiC/4.6FeCr powders obtained after 50 h of the ball milling was investigated by DSC analysis conducted with heating rates (*k*) of 7, 8, 9, and 10 °C/min and presented in Figure 4. All the scans revealed single endothermic events related to the decomposition of MgH_2_ phase. While the peak height increased proportionally with increasing heating rates, the peak temperatures (*T*_p_) were significantly shifted to the higher temperature side upon increasing the heating rates from 7 °C/min to 10 °C/min, as shown in Figure 4. The peak decomposition temperature performed at a heating rate of 10 °C/min was 658 K (385 °C). When comparing this value with that (441 °C) obtained for nanocrystalline MgH_2_ powders [12], one can say that doping MgH_2_ with 5.2 wt. % TiC/4.6 wt. % FeCr powders led to destabilizing the metal hydride phase and decreasing the decomposition temperature by 56 °C.

**Figure 5:**
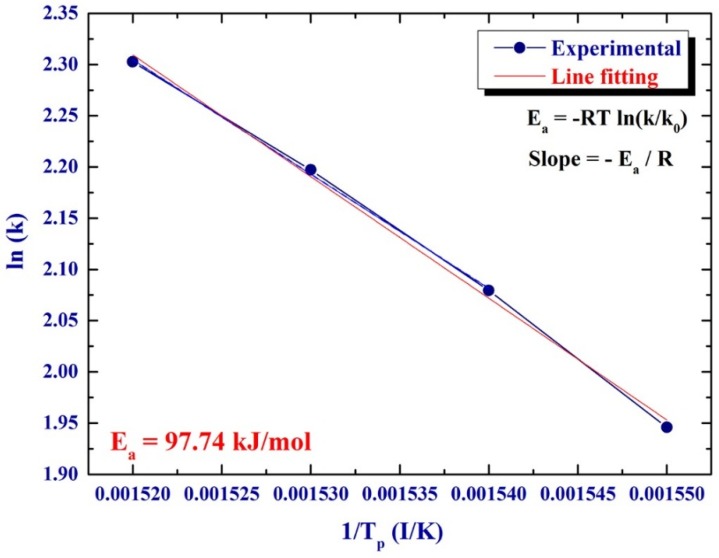
Arrhenius plot displayed the natural logarithmic values of the heating rates (*k*)* versus* the inverse of the peak temperature (1/*T*_p_) denoted in the DSC curves of Figure 4.

The improved dehydrogenation kinetics in a helium gas atmosphere was investigated by calculating the activation energy (*E*_a_) of the decomposition reaction. In the present work, the activation energy for dehydrogenation of MgH_2_ doped with TiC/FeCr was calculated according to the Arrhenius Equation:

*E*_a_ = −*RT* ln(*k*)

where *k* is a temperature-dependent reaction rate constant, *R* is the gas constant, and *T* is the absolute temperature. The value *E*_a_ of the reaction was determined by measuring the decomposition the *T*_p_ corresponded to the different heating rates (*k*) and then plotting ln(*k*)* versus* 1/*T*_p_, as shown in Figure 5. A best fit for the results was calculated by the least-square method. It follows from Figure 5 that all data points lie closely on the same straight line. The *E*_a_ of 97.74 kJ/mol was obtained from the slope of line (−*E*/*R*). This value, which is far below than that one (146.53 kJ/mol) calculated for pure MgH_2_ powders [12], indicating a significant improvement of the dehydrogenation kinetics of the MgH_2_ upon doping with 5.2TiC/4.6FeCr.

**Figure 6:**
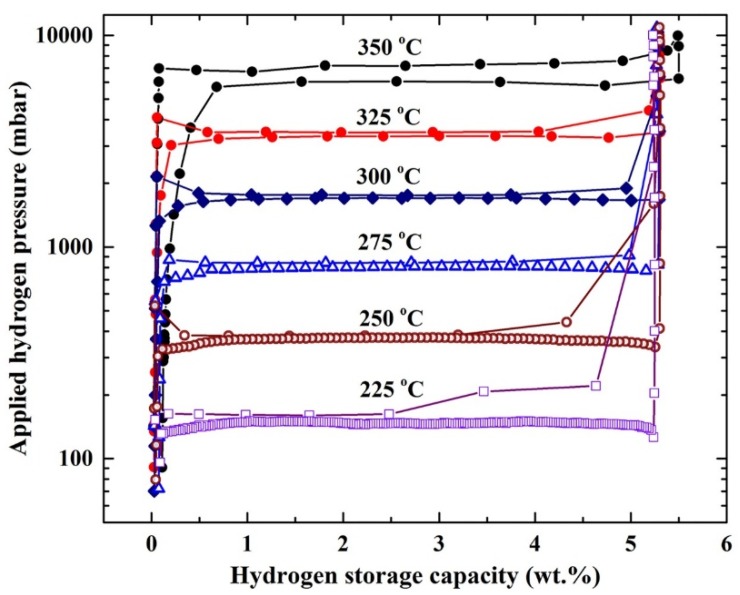
Pressure-composition-temperature (PCT) curves of ball-milled MgH_2_/5.2 TiC/4.6 FeCr nanocomposite powders obtained after 50 h at different temperatures of 225, 250, 275, 300, 325, and 350 °C.

**Figure 7:**
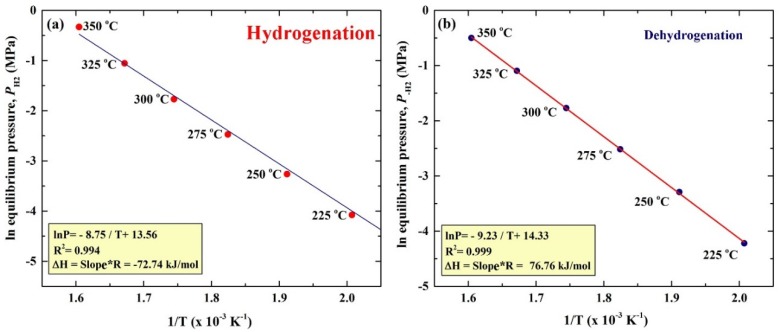
van’t Hoff plot of the plateaus shown in Figure 6 for the (**a**) hydrogenation, and (**b**) dehydrogenation of ball-milled MgH_2_/5.2TiC/4.6FeCr nanocomposite powders obtained after 50 h.

The pressure-composition temperature (PCT) relations of ball-milled MgH_2_/5.2TiC/4.6FeCr nanocomposite powders obtained after 50 h were volumetrically investigated by Sievert’s approach at different temperatures of 225, 250, 275, 300, 325, and 350 °C, as elucidated in Figure 6. A single reversible hydrogenation/dehydrogenation cycle was developed for each applied temperature. The presence of clear hydrogenation plateaus can be seen in the range between 0.25 and 5.25 wt. % H_2_ at temperatures ranging between 275 and 350 °C, as shown in Figure 6. However, the hydrogen uptake plateau was visible only in the range of 0.25–2.5 and 0.25–3.25 wt. % H_2_, at temperatures of 225 °C and 250 °C, respectively. On the other hand, smooth plateaus of hydrogen release were characterized in the whole hydrogen concentrations range (0.25–5.25 wt. % H_2_) for all applied temperatures, as presented in Figure 6. The hydrogen equilibrium pressure measurements were used in the present study to investigate the heat of hydrogen absorption, using van’t Hoff equation:

(1) $$\ln(\frac{P_{eq}}{P_{o}})=-\frac{\text{∆H}}{\text{RT}}+\frac{\text{∆S}}{\text{R}}$$

where $P_{eq}$ is the hydrogen pressure under equilibrium at a given specific temperature, *T*; *P*_0_ is a reference pressure of 1 bar; *R* is the gas constant (0.0083145 J/K.mol); Δ*H* is the molar enthalpy of metal hydride formation (MgH_2_); and Δ*S* is the entropy of absorption. Thus, Δ*H* can be directly calculated from plotting the natural log of each $P_{eq}$ point* versus* the corresponding 1/*T*, as shown in Figure 7a. In the present work, the calculated ∆*H* and ∆*S* for MgH_2_ doped with 5.2TiC/4.6FeCr was −72.74 kJ/mol and 112.79 J/mol H_2_/K, respectively.

The strength of Mg–H bonds, which can be expressed by the enthalpy of decomposition can be calculated by van’t Hoff approach, using the equilibrium dehydrogenation pressure in the PCT measurements. A van’t Hoff plot illustrating the relationship between ln(*P*) and 1/*T* for the decomposition of MgH_2_ powders doped with 5.2TiC/4.6FeCr is shown in Figure 7b. Both of ∆*H* and ∆*S* were directly calculated from the slope of the curve presented in Figure 7b and found to be 76.76 kJ/mol and 119.15 J/mol H_2_/K, respectively. Comparing these values with those reported by Reilly (77.4 kJ/mol, 138.3 J/mol H_2_/K) [22], and Klose (81.86 kJ/mol, 146.1 J/mol H_2_/K) [23], one can say that long-term ball milling led to the formation of homogeneous nanocomposite MgH_2_/5.2TiC/4.6FeCr powders, destabilizing the chemically stable phase of MgH_2_, implied by the obvious increase in the ∆*H* of decomposition. Until recently, it was believed that ∆*S* has a constant value of about 130 J/mol H_2_/K [24]. It has been suggested by Zhao-Karger* et al.* [24] that ∆*S* of the dehydrogenation process can be varied based on the MgH_2_ particle size. Based on the *ab initio* Hartree-Fock and density functional theory calculations shown by Wagemans* et al.* [25], magnesium hydride becomes less stable with decreases in the cluster size to less than 20 atoms. Accordingly, and based on that study, the ∆*H* of hydrogen desorption decreases significantly when the grain size is smaller than 1.3 nm [25].

**Figure 8:**
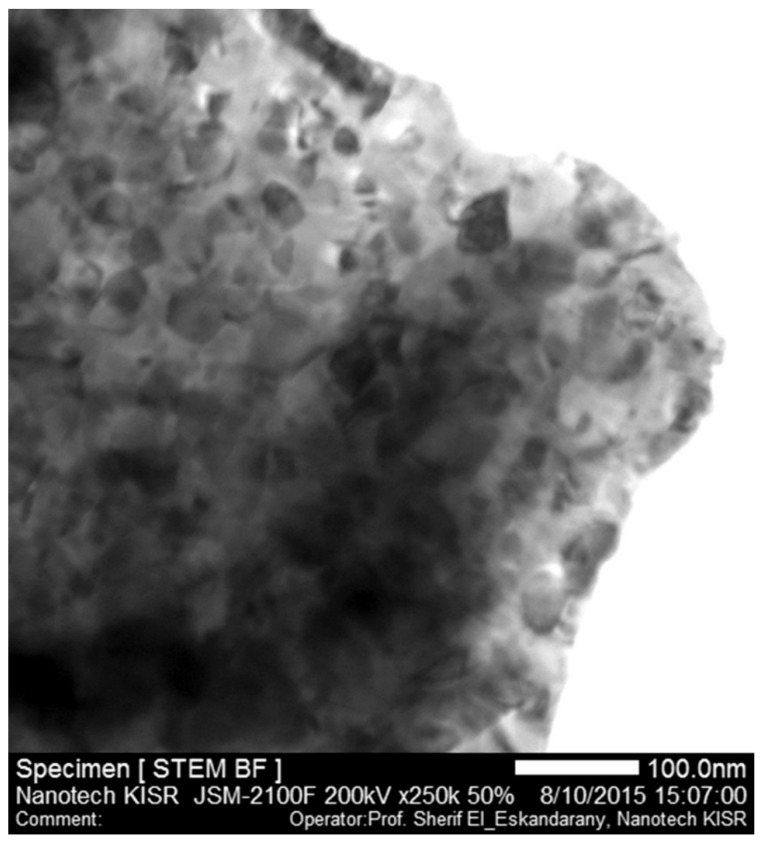
STEM/BF image of ball-milled MgH_2_/5.2TiC/4.6FeCr nanocomposite sample after achieving the PCT hydrogenation/dehydrogenation measurements under hydrogen gas pressure and temperatures ranging between 0 and 10 bar, and 225 and 350 °C, respectively.

Figure 8 displays the STEM/BF image of the ball-milled nanocomposite sample after the PCT hydrogenation/dehydrogenation measurements under hydrogen gas pressure and temperatures ranging between 0 and 10 bar, and 225 and 350 °C, respectively. Obviously, the sample maintained its nanocrystalline structure ranging between 18 and 67 nm for MgH_2_ matrix (light gray-scale particles) and 8 and 27 nm for TiC (dark particles), as shown in Figure 8. We should emphasize that the as-prepared ultrafine powders in the present study with their nanostructured grains facilitated better hydrogen desorption and shortened the diffusion distance required to accomplish a complete dehydrogenation process. In addition, TiC refractory nanoparticles acted as grain growth inhibitors maintaining the MgH_2_ particles, especially when the samples were subjected to the high temperature side (300–350 °C) during the PCT analysis.

**Figure 9:**
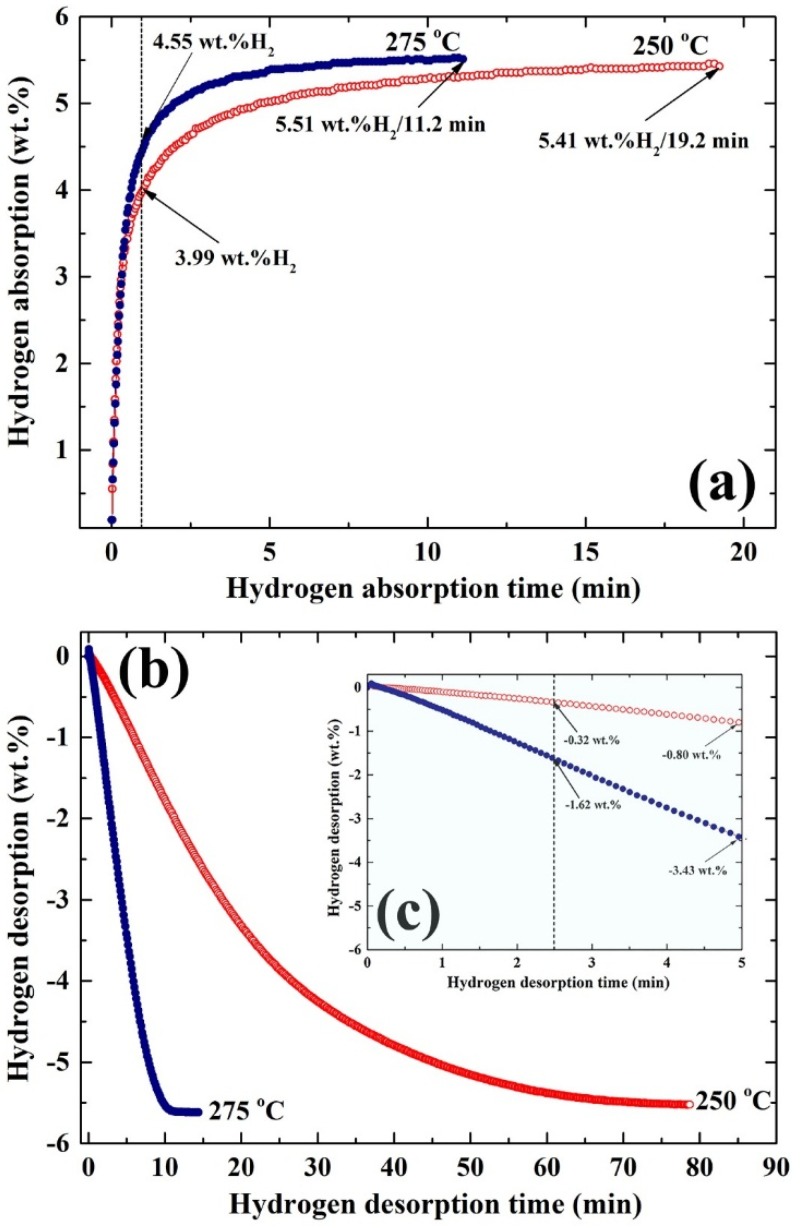
Effect of temperature and time on the (**a**) hydrogenation; and (**b**) dehydrogenation kinetics of nanocomposite MgH_2_/5.2TiC/4.6FeCr powders obtained after ball milling for 50 h. The dehydrogenation kinetics measured at 250 °C (open symbols) and 275 °C (closed symbols) after 5 min of desorption are presented in Figure 9c.

Figure 9 displays the temperature effect on the hydrogen absorption (a) and consequence desorption (b,c) kinetics of nanocomposite MgH_2_/5.2TiC/4.6FeCr powders obtained after 50 h of the ball milling. In general, the synthesized nanocomposite powders showed excellent potential for absorbing hydrogen gas in a short time at temperatures ranging from 250 to 275 °C under pressure ranging from 100 mbar to 8 bar, as shown in Figure 9a. After 1 min, the powders examined at 250 and 275 °C were able to uptake 3.66 and 4.55 wt. % H_2_, respectively as elucidated in Figure 9a. After 11.2 min of the absorption, the sample examined at 275 °C reached its saturated value with hydrogen storage reaching 5.51 wt. %. In contrast, 19.2 min was required for the sample examined at 250 °C to absorb 5.41 wt. % H_2_, as shown in Figure 9a.

The corresponding desorption kinetics of the nanocomposite powders investigated at 250 °C and 275 °C are shown in Figure 9b,c. The powders examined at 275 °C showed excellent desorption kinetics, indexed by the relatively short time (~10 min) required to release about 5.51 wt. % of hydrogen, as shown in Figure 9b. The sample examined at this temperature desorbed 1.62 wt. % of hydrogen within a short desorption time of 2.5 min, as shown in Figure 9c. At this applied temperature, the sample released about 3.43 wt. % of its hydrogen storage capacity after 5 min of desorption, as elucidated in Figure 7c. In contrast to such fast desorption kinetics achieved at 275 °C, the sample examined at 250 °C showed a slow dehydrogenation behavior, indexed by the long time required to release its full hydrogen content (~5.5 wt. %), 79 min, as shown in Figure 9b. After 2.5 and 5 min of desorption conducted at 250 °C (Figure 9c), the sample was unable to release more than 0.32, and 0.80 H_2_ wt. %, respectively, as presented in Figure 9c. Aside from the particle size effect on the ∆*H* and ∆*S* of hydrogen desorption for MgH_2_, the dehydrogenation temperature decreased from 400 °C in bulk MgH_2_ to be 250–275 °C, when the crystallite size of MgH_2_ was less than 10 nm in diameter (Figure 2a).

**Figure 10:**
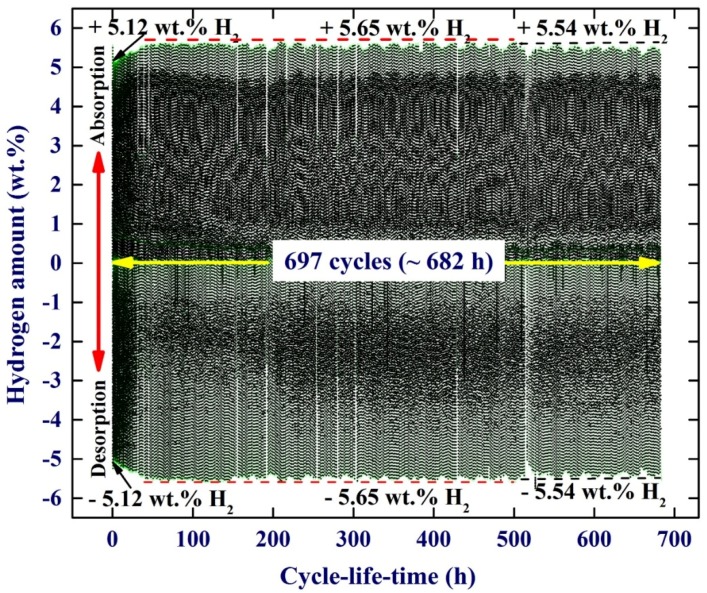
(**a**) Hydrogenation and consequent dehydrogenation curves of 697 complete cycles performed within 682 h for nanocomposite MgH_2_/5.2TiC/4.6FeCr powders obtained after 50 h of ball milling. The hydrogen absorption and desorption processes were achieved at a constant temperature of 275 °C with an applied pressure of 100 mbar/8 bar.

Apart from the fast kinetics of hydrogenation/dehydrogenations characterizations shown by MgH_2_/5.2TiC/4.6FeCr ternary system, the cyclic-reversibility of the fabricated nanocomposite powders examined at 275 °C under repeated hydrogenation/dehydrogenation pressure of 0/8 bar was investigated. Figure 10 shows the cycle-life-time performed at 275 °C for the nanocomposite powders obtained after 50 h of ball milling. Obviously, this new nanocomposite system exhibits excellent cyclic-reversible properties, indexed by its high cyclic stability without failure, even after about 682 h (679 cycles), as shown in Figure 10. Comparing the number of cycles achieved at 275 °C by this nanocomposite system with those performed in MgH_2_/Mn_3.6_Ti_2.4_, 1000 cycles/275 °C [16], MgH_2_/5Ni5Nb_2_O_5_, 180 cycles/250 °C [26], MgH_2_/5Fe 47 cycles/300 °C [27], and MgH_2_/10Co 350 °C [25] systems, one can consider the MgH_2_/TiC/FeCr system as one of the most stable and capable MgH_2_-based nanocomposite systems used for hydrogen storage applications.

## 4. Conclusions

Nanocrystalline MgH_2_ powders were synthesized by reactive ball milling of pure Mg powders, using a high-energy ball mill operated at 250 rpm under 50 bar of hydrogen atmosphere. The as-synthesized MgH_2_ powders obtained after 200 h of ball milling were contaminated by about 2.2 wt. % of FeCr. The powders were doped with TiC ultrafine powders, which were already contaminated with 2.4 wt. % FeCr, and then ball milled for 50 h. Significant improvements in the hydrogenation/dehydrogenation kinetics of MgH_2_ doped with 5.2TiC/4.6FeCr were achieved. Such improvements are attributed to the presence of FeCr content that played an important role in splitting the H_2_ molecules and facilitating proper hydrogen diffusion into the Mg matrix. In addition, ball milling the MgH_2_ powders with refractory TiC nanopowders led to further grain refining of the metal hydride phase, enabling fast hydrogen absorption/desorption processes. Moreover, the hard TiC phase inhibited grain growth, allowing to maintain the nanocrystallinity of MgH_2_ powders during repeated hydrogenation/dehydrogenation cycles that extended to 697 cycles without failure or degradation.
